# Targeting BRD4—A Promising Therapeutic Option for Glioblastoma?

**DOI:** 10.3390/ijms27052268

**Published:** 2026-02-28

**Authors:** Maria Lindner, Dagmara Lisińska, Anna Kędzierzyńska, Aleksandra Majchrzak-Celińska

**Affiliations:** 1 The Student Scientific Society Biomolekularni, Poznan University of Medical Sciences, 60-806 Poznań, Poland; lindner.maria02@gmail.com (M.L.); lisinskad@gmail.com (D.L.); annked04@gmail.com (A.K.); 2 Department of Pharmaceutical Biochemistry, Poznan University of Medical Sciences, Rokietnicka 3, 60-806 Poznań, Poland

**Keywords:** glioblastoma, glioma, BRD4, bromodomain and extra-terminal (BET) proteins, epigenetic dysregulation, BET inhibitors, BRD4 degraders, targeted therapy, therapeutic resistance

## Abstract

Epigenetic dysregulation is increasingly recognized as a key driver of glioblastoma (GBM), with bromodomain-containing protein 4 (BRD4) emerging as a critical regulator of tumor malignancy. GBM is an aggressive brain tumor marked by diffuse infiltration, a population of stem-like cells and multiple resistance mechanisms, which together render it largely incurable. Standard treatment, consisting of surgical resection followed by radiotherapy and temozolomide chemotherapy, confers only limited therapeutic benefit, while a member of the bromodomain and extra-terminal (BET) family, BRD4, regulates transcriptional programs essential for oncogene activation, chromatin stability and glioma cell survival. Its expression is markedly elevated in GBM relative to normal brain tissue, implicating BRD4 in tumor initiation, progression and therapeutic resistance. Recent advances have enabled the development of selective BRD4 inhibitors and degraders capable of penetrating the blood–brain barrier and preferentially targeting glioma cells. Preclinical and early-phase clinical studies indicate that these agents suppress tumor growth and may enhance the efficacy of existing treatments. Although BRD4 clearly influences glioma progression and modulates key oncogenic pathways, the precise mechanisms underlying BRD4-driven gliomagenesis remain only partially understood. Ongoing research continues to advance knowledge of its multifaceted functions. This review summarizes current knowledge on BRD4 in GBM, evaluates emerging BRD4-targeted therapeutic strategies and outlines major challenges and future directions for clinical translation.

## 1. Introduction

Glioblastoma (GBM) is one of the most severe tumors and represents the most common primary neoplasm of the brain and central nervous system (CNS), with an annual incidence ranging from 1.9 to 9.6 cases per 100,000 people [1,2]. Derived from astrocytic glial cells, it is classified by the World Health Organization (WHO) as a grade 4 glioma, the highest grade of malignancy [1]. Most patients are diagnosed in their 60s, with incidence peaking in individuals over 75 years of age [3]. GBM is characterized by rapid proliferation and marked invasiveness, which frequently results in a poor prognosis [4]. Despite aggressive treatment, including gross total resection followed by combined temozolomide (TMZ) chemotherapy and radiotherapy (RT), patient outcomes remain unfavorable. Postoperative survival typically does not exceed two years, and the five-year survival rate remains around 5.8% [5]. The median overall survival for GBM patients is approximately 15 months [6].

According to the current understanding, the persistence of tumor growth, therapeutic resistance and recurrence is primarily attributed to a heterogeneous tumor mass containing a small subpopulation of glioma stem cells (GSCs), distinguished by their self-renewal capacity and ability to maintain stemness [7,8,9]. This has prompted ongoing efforts to identify novel molecular targets to improve therapeutic efficacy and patient outcomes.

Both genetic and epigenetic alterations contribute to GBM pathogenesis. At the genetic level, isocitrate dehydrogenase (*IDH*)-wildtype GBM, typically lacks *IDH* mutations and frequently shows changes in the *TERT* promoter, chromosomes 7, 10 and Epidermal Growth Factor Receptor (*EGFR*). Moreover, mutations in *PTEN* enhance its stability and nuclear localization, promoting tumor growth, while *TP53* gain-of-function mutations are linked to poorer prognosis and reduced TMZ sensitivity [10,11,12,13]. In contrast, *IDH*-mutant gliomas, including oligodendrogliomas and astrocytomas, exhibit distinct biological characteristics, demonstrating slower clinical progression and improved overall prognosis [14].

Epigenetic modifications are defined as mitotically heritable changes in gene activity that modulate gene expression without altering the primary DNA sequence [15,16]. These mechanisms, which include DNA methylation, histone modifications, chromatin remodeling, and non-coding RNA expression, are fundamental for regulating chromatin structure and determining cell fate [17,18]. Abnormalities within these pathways are widely recognized as major contributors to cancer initiation and progression, acting alongside genetic mutations to drive tumorigenesis [19]. Disruptions such as altered DNA methylation patterns or abnormal histone marks can promote genomic instability and facilitate the acquisition of malignant characteristics by cancer cells [20].

Importantly, unlike genetic mutations that are often permanent, epigenetic aberrations exhibit an inherent reversibility. This feature carries substantial therapeutic potential and sets them apart from classical genomic alterations [21]. The plasticity of epigenetic regulation creates valuable opportunities for pharmacological intervention in oncology. As a result, the targeted modulation of epigenetic mechanisms through strategies such as drug combination therapies or advanced epigenetic editing has become an area of considerable research interest [21]. Owing to their dynamic and reversible nature, components of epigenetic machinery are increasingly viewed as highly promising targets for the development of novel cancer therapeutics [16,22].

Prominent among the components of this machinery are the bromodomain and extra-terminal domain (BET) proteins (BRD2, BRD3, BRD4 and BRDT). These proteins function as epigenetic “readers” that bind acetylated lysine residues on histones, thereby enabling transcriptional complex assembly and gene expression [23,24]. In the context of GBM, BET proteins are significantly overexpressed compared to normal brain tissue [25]. Their activity is closely linked to the regulation of oncogenes and pathways involved in cell proliferation, apoptosis, and differentiation, highlighting their potential as therapeutic targets in GBM treatment [26].

Although multiple BET family members are expressed in GBM, their functional roles are not equivalent. Among these, bromodomain-containing protein 4 (BRD4) consistently emerges as the principal BET protein driving GBM progression, primarily through its epigenetic regulation of oncogenic transcription programs, cell cycle progression, and stem-like properties of tumor-initiating cells [27,28].

In contrast, BRD2 and BRD3 appear to play a context-dependent or auxiliary role. Functional analyses demonstrate that BRD4 loss more strongly suppresses proliferation and clonogenicity than loss of BRD2 or BRD3, highlighting its unique role in tumor aggressiveness [29,30]. BRD3’s role is weaker and less consistent, though it may influence enhancer activity under specific conditions [30]. BRDT, the fourth member of the BET family, shows testis-restricted expression and has not been implicated in GBM. It is therefore typically excluded from studies of this type [31].

Structurally, BRD4 is characterized by two tandem N-terminal bromodomains (BD1 and BD2) which serve as recognition pockets that specifically bind to histone acetylation marks, particularly at promoter and enhancer regions [32,33,34]. Mechanistically, BRD4 acts as a molecular scaffold that facilitates transcriptional activity by recruiting key regulatory complexes [35,36]. A vital function of BRD4 is the recruitment and activation of the positive transcription elongation factor (P-TEFb) [32,37]. This interaction is essential for releasing paused RNA Polymerase II (Pol II) from the promoter region, leading to its phosphorylation and the activation of productive transcriptional elongation [25,38].

## 2. Structure and Biological Function of BRD4

The BET protein family consists of four members—BRD2, BRD3, BRD4 and BRDT [39,40]—which share a characteristic structural organization defined by specific conserved domains [27]. These proteins act as epigenetic “readers” by utilizing two tandem N-terminal bromodomains (BD1 and BD2) to recognize acetylated lysine residues on histones and other nuclear proteins [27,28]. While they share a general domain structure, including an extra-terminal (ET) domain for recruiting co-activators, there are significant functional and structural differences between them [28,40,41,42,43]. BRD2, for instance, acts as an atypical protein kinase and is indispensable for the G1/S phase transition through E2F1 and E2F2 protein activation, while BRD3 specifically interacts with the acetylated sites on the *GATA1* transcription factor to orchestrate the gene expression programs essential for the erythroid lineage and megakaryocyte development [44,45]. In contrast, BRDT is critical for male germline development and meiotic division [45]. A key structural divergence is that only BRD4 and BRDT possess a C-terminal motif (CTM), also called the C-terminal domain (CTD), which is required for recruiting the P-TEFb to regulate gene expression [42]. Within the context of GBM, BRD4 is the most significant player, as it uniquely binds to super-enhancers to drive oncogenic transcription and constitutes the central subject of this review [27,42].

The molecular architecture of BRD4 is fundamental to its biological function. The structure of the BD1 and BD2 domains, consisting of four alpha-helices separated by variable loop regions, folds to create a deep, hydrophobic cavity [27,39,43]. The hydrophobic pocket is structured to specifically recognize and bind to acetylated lysine (Kac) residues [41]. This allows BRD4 to bind to acetylated histones (e.g., H3K27ac, H4K5ac, H4K12ac) and non-histonic proteins (e.g., RelA subunit of nuclear factor κB [NF-κB], TWIST, and *GATA1*). This interaction attaches BRD4 to discrete locations on acetylated chromatin [28,43]. While both BD1 and BD2 recognize acetylated lysine, they have distinct functions. BD1 is considered critical for chromatin binding, while BD2 is involved in mediating the recruitment of other BET proteins [34]. Following the bromodomains is the ET domain [27,28,43]. Structurally, this domain adopts a compact fold composed of three alpha-helices and a loop [27]. This domain functions as a protein–protein interaction scaffold [42] that facilitates the recruitment of transcriptional cofactors and enhances the transcription process [45]. The ET domain recruits various histone modifiers, such as the arginine demethylase (JMJD6) and the lysine methyltransferase (NSD3) [42], as well as ATP-dependent nucleosome-remodeling enzymes [43].

**Figure 1:**
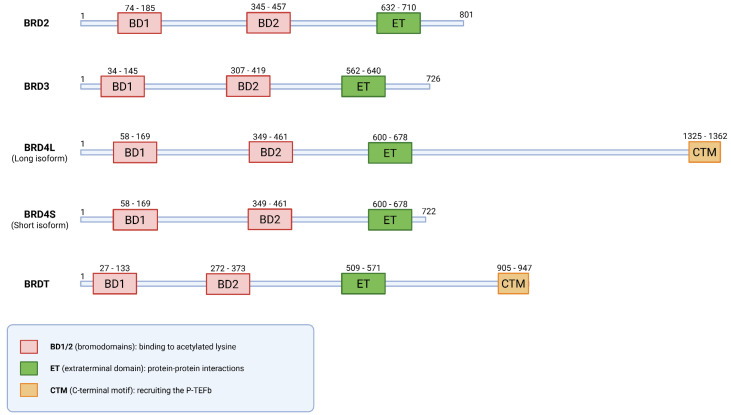
Structural overview of BET family proteins. Created in BioRender. Krajka-Kuźniak V. (2026), https://BioRender.com/h73kn2p (accessed on 27 January 2026).

BRD4 exists in two isoforms (Figure 1), a long (BRD4L) and a short (BRD4S) form, which results from alternative splicing. Both isoforms contain the two bromodomains (BD1 and BD2) [37,46]. A key difference between them is the CTM, which is unique to the BRD4L isoform and is not present in BRD4S [41,46]. This CTM is responsible for recruiting P-TEFb [27,45,46], which is a heterodimer of cyclin-dependent kinase 9 (CDK9) and cyclin T1 [43,47].

**Figure 2:**
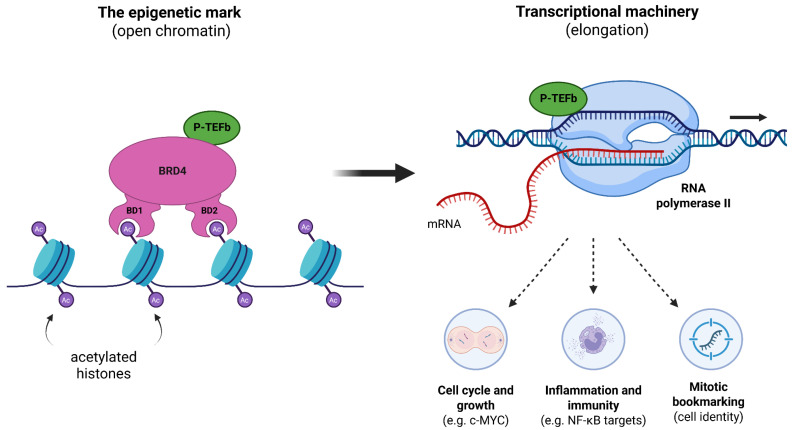
The role of BRD4 as an epigenetic reader and transcriptional scaffold. BRD4 functions as an epigenetic reader and molecular scaffold that links acetylated, transcriptionally permissive chromatin to the machinery required for productive gene expression. Through its bromodomains, BRD4 recognizes acetylated histone lysines and stabilizes open chromatin. BRD4 subsequently recruits and coordinates co-activator complexes, including P-TEFb, which phosphorylates RNA Pol II and associated elongation factors to promote the transition from promoter proximal pausing to productive transcriptional elongation. Through this central regulatory role, BRD4 governs key transcriptional programs controlling cell cycle progression, inflammatory and immune signaling, and the maintenance of cell identity via mitotic bookmarking. Created in BioRender. Krajka-Kuźniak V. (2026), https://BioRender.com/st29psl (accessed on 27 January 2026).

BRD4’s structure enables it to function as a key transcriptional regulator, involving it in numerous biological activities including cell cycle progression, cell growth, embryonic development, and maintaining genome stability [28,36,45]. The most well-studied function of BRD4 is its role in transcriptional elongation. BRD4 acts as a scaffold that recruits transcription factors and co-activators to target gene sites [36,41,48]. This mechanism is initiated when BRD4 binds to acetylated histones at active promoters and enhancers [27,48]. Once bound, the CTM of BRD4 (specifically the long isoform) recruits the P-TEFb complex [45,49]. This interaction is crucial because it prevents P-TEFb from associating with the 7SK/HEXIM ribonucleoprotein complex, which normally sequesters P-TEFb in an inactive state [43]. By recruiting active P-TEFb, BRD4 facilitates the activation of RNA Pol II [49]. Specifically, BRD4 helps to release Pol II from a “promoter proximal pause”, a key regulatory checkpoint [38,46]. The recruited P-TEFb then phosphorylates Pol II, allowing it to transition into a productive state and begin transcriptional elongation [41,45,48] (Figure 2).

BRD4 exerts a pronounced effect not just at standard promoters, but also at super-enhancers (SEs). SEs are large clusters of regulatory regions that control genes related to cell identity and disease [41]. BRD4 recognizes, binds to, and activates these SEs. In disease states, BRD4 extensively accumulates on SEs, often together with mediator complexes [38,50,51]. This high-level accumulation of BRD4 drives the significant overexpression of key oncogenes like *MYC* [27,49] and orchestrates transcriptional programs in GBM involving genes such as *RUNX1*, *BHLHE40*, *BCL3*, *FOSL2* [52], *EGFR*, and *SOX2* [50]. The functional significance of this regulation is demonstrated by the marked enhancement in GBM cell proliferation observed in vitro and the subsequent advancement of tumor growth in vivo, which is essential to fully understand the molecular mechanisms and the complex internal structure that drive GBM progression [50,52].

Apart from transcriptional elongation, BRD4 plays a role in several other critical cellular processes. BRD4’s association with acetylated chromatin is important for maintaining higher-order chromatin structures and defining transcriptional limits [41,53]. Interfering with BRD4 results in chromatin decondensation and fragmentation. It also functions as a “histone chaperone”, helping RNA Pol II move through acetylated nucleosomes and ensuring the elongation of nascent transcripts [23,38]. BRD4 possesses its own intrinsic histone acetyltransferase (HAT) activity, allowing it to directly acetylate histones H3 and H4 to promote transcriptional elongation [46,54]. This enzymatic capability is a specialized function primarily attributed to the long isoform of the protein (BRD4L). While BRD4 can form complexes with traditional HATs like p300/CBP to enhance their activity [41,55], it also exerts an independent enzymatic effect through a distinct mechanism that facilitates the eviction of nucleosomes from chromatin [54]. This HAT activity is specifically linked to the CTD, a structural element absent in the short isoform, which lacks the capacity for significant HAT activity [54,55]. Finally, BRD4 is an important cell cycle regulatory protein [45,56,57]. It remains bound to chromosomes during mitosis, when most other factors are released [23], acting as a “mitotic bookmark” to mark genes for transcription in the subsequent G1 phase. Deficiency of BRD4 leads to aberrant mitosis and cytokinesis failure [36].

Although indispensable for physiological cellular processes, the aberrant overexpression or dysregulation of BRD4 is implicated in the etiology of numerous pathologies, most notably cancer such as GBM [27,28,36]. Functioning as a transcriptional regulator [28], BRD4 contributes to cancer progression by influencing oncogene transcription via SEs [49]. Consequently, the pharmacological suppression of BRD4 activity has been demonstrated to potently attenuate cancer cell proliferation while inducing both cell cycle arrest and apoptosis [27].

## 3. BRD4 in Glioblastoma

**Figure 3:**
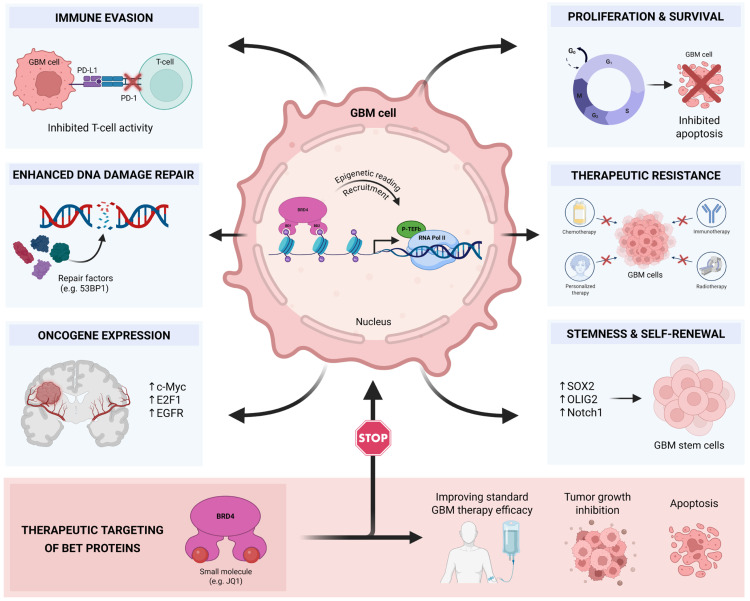
BRD4 as a key driver of GBM progression and a therapeutic target. By regulating transcriptional programs involved in immune evasion, DNA damage repair, oncogene expression, proliferation, therapeutic resistance and stem cell self-renewal, BRD4 promotes GBM aggressiveness and offers opportunities for therapeutic intervention. Created in BioRender. Krajka-Kuźniak V. (2026), https://BioRender.com/7wl9yv6 (accessed on 14 February 2026).

Given the structural diversity within the BRD family, selective targeting of specific domains—such as BD1 or BD2 within BET proteins—as well as other members including BRD4 or testis-specific BRDT, is of considerable importance. Recent efforts have led to the development of BD1 and BD2-selective inhibitors, demonstrating that differential domain targeting can result in distinct biological outcomes. Moreover, selective degraders of BRD4 have shown enhanced functional specificity compared to pan-BET inhibitors. These examples highlight the therapeutic value of developing inhibitors or degraders with defined selectivity profiles across individual BRD family members [58,59,60,61,62,63]. BRD4 has emerged among them as an important epigenetic regulator and transcriptional co-activator in GBM. BRD4 binds to acetylated lysine residues on histone tails, helps the cell’s transcription tools reach promoters and SEs, and sustains the expression of genes essential for proliferation, DNA repair and cell survival [27,28]. Through its role in maintaining oncogenic transcriptional networks, BRD4 is positioned at the center of GBM’s transcriptional addiction, cellular plasticity, and therapeutic resistance. Mechanistically, BRD4 localizes to SEs, which are large clusters of enhancers that control the expression of genes essential for cell identity and oncogenic behavior. In GBM, these SEs are enriched in parts that control *MYC*, *SOX2*, *OLIG2*, and other regulators of proliferation and stemness. By maintaining the open chromatin state and recruiting P-TEFb, BRD4 ensures continuous RNA Pol II elongation at these oncogenic parts. Consequently, BRD4 activity underpins the transcriptional networks that promote rapid tumor growth, therapy resistance and the maintenance of GSC populations, making it a compelling target for therapeutic strategy [27,28,64,65] (Figure 3).

Accumulating evidence supports BRD4 as an active tumorigenic driver. Functional knockdown of BRD4 via RNA interference or CRISPR in GBM cell lines such as U251 significantly reduces proliferation, induces apoptosis and changes global gene expression in models, particularly in pathways regulating the cell cycle, chromatin structure and DNA replication [65]. Pharmacologic inhibition of BRD4 with BET inhibitors such as JQ1, I-BET151 or next-generation degraders such as GNE987 suppresses GBM cell growth, blocks clonogenicity, and induces apoptosis [30,66].

Clinically, BRD4 is markedly overexpressed in GBM relative to normal brain tissue. Studies of TCGA and CGGA datasets show that high BRD4 levels are linked to more advanced tumors, fewer immune cells in the tumor, and shorter patient survival [67]. In a recent bioinformatic and clinical study, BRD4 protein levels were significantly higher in GBM than in the normal brain, and high expression was associated with lower infiltration of CD8+ T cells, macrophages, and neutrophils [68]. These findings suggest that BRD4 not only drives tumor proliferation but also contributes to an immunosuppressive microenvironment that worsens prognosis.

BRD4 promotes GBM progression through several interrelated pathways. First, BRD4 binds to enhancers controlling critical oncogenes, such as *c-MYC*, E2F1, and a long non-coding RNA, *HOX* transcript antisense RNA resistance (HOTAIR), sustaining high-amplitude transcription through RNA Pol II phosphorylation and elongation [49]. Second, BRD4 influences the DNA damage response and therapeutic ncRNA. Its inhibition attenuates expression of DNA repair and resistance genes, notably O-6-methylguanine-DNA methyltransferase (*MGMT*) gene, sensitizing GBM cells to TMZ and RT [69,70]. Third, BRD4 maintains GSC identity and self-renewal by regulating stemness pathways, such as the Notch1 signaling axis, thereby sustaining tumor heterogeneity and recurrence [64]. Fourth, BRD4 acts as an immunomodulator within the GBM microenvironment. It activates Programmed Death Ligand 1 (PD-L1) by binding to its promoter region, promoting immune evasion and suppressing antitumor immunity [71]. High BRD4 expression is associated with reduced immune cytotoxic cell infiltration and an immunosuppressive cytokine environment, further underscoring its role in tumor immune escape [67]. Finally, there are BRD4 functions, such as RNA binding, protein scaffolding and susceptibility to proteasomal degradation through PROTACs, which broaden its influence beyond chromatin interaction and offer novel therapeutic entry points [72,73].

GSCs, also referred to as glioma-initiating cells (GICs), represent a subpopulation of GBM cells characterized by self-renewal, multipotency, and strong tumorigenic potential, thereby sustaining the heterogeneity and malignant progression of GBM [64,74]. These cells have been implicated in tumor initiation, therapeutic resistance and recurrence, as they possess the ability to survive chemotherapy and RT, leading to treatment failure and tumor relapse [64,75,76]. Importantly, their stem-like nature is maintained by specific molecular pathways and epigenetic mechanisms that promote continuous proliferation and survival within specialized niches as perivascular and hypoxic microenvironments [77].

At the molecular level, BRD4 has emerged as a key regulator of GSC stemness and tumorigenicity. It exerts its function by binding to the promoters of oncogenic drivers, such as Notch1 and miR-142-5p, thereby enhancing the transcription of genes that maintain the stem cell phenotype [64,78,79,80]. Notch1 serves as a central signaling mediator controlling cell determination, proliferation, and differentiation in both normal neural stem cells and GSCs. In GSCs, abnormal activation of the Notch1 pathway maintains their undifferentiated state and promotes tumorigenic potential. By transcriptionally increasing Notch1 expression, BRD4 strengthens these oncogenic processes, supporting GSC self-renewal and promoting resistance to therapy [81]. Moreover, BRD4 supports the self-renewal capacity of GSCs by modulating major oncogenic signaling cascades, such as the VEGF/PI3K/AKT and Wnt/β-catenin pathways [79,80]. Inhibition of BRD4 has been shown to suppress GSC proliferation and self-renewal, resulting in reduced tumor growth in intracranial GBM models [64]. Such a broad regulatory role of BRD4 in maintaining stemness and activating oncogenic signaling suggests that it also contributes to the development of therapeutic resistance in GBM.

Recent studies identify BRD4 as a crucial epigenetic regulator contributing to the development of therapeutic resistance in GBM [29,70]. The transcriptional programs driven by BRD4 enable tumor cells to adapt to chemotherapeutic and radiotherapeutic stress [29]. In particular, BRD4 activity has been associated with reduced sensitivity of GBM to TMZ, largely through transcriptional regulation of *MGMT*, a key determinant of alkylating drug resistance, by maintaining its association with promoter and enhancer regions that support RNA Pol II recruitment and transcriptional elongation [70,82]. Beyond *MGMT* regulation, BRD4 also reinforces survival signaling pathways, including Notch1, and redox balance, thereby enabling glioma cells to withstand the cytotoxic and oxidative stress induced by chemotherapy [83].

In the context of radiotherapy (RT), BRD4 contributes to resistance by controlling transcriptional programs that support DNA double-strand break repair and sustaining SE-driven expression of survival-associated genes such as type I collagen (*COL1A1*), thereby promoting radioresistance and maintaining clonogenic survival of GBM cells [49,69].

In addition to its direct effects on DNA repair and survival pathways, BRD4 modulates stress-responsive oncogenic signaling. Under therapeutic stress, it enhances the transcription of pro-survival genes regulated by MYC, NF-κB and STAT3, consequently establishing an adaptive transcriptional state that allows GBM cells to survive cytotoxic insults. This epigenetically driven plasticity supports a transient, drug-tolerant phenotype that can evolve into stable resistance during treatment [71,84,85]. Furthermore, emerging evidence indicates that BRD4-mediated upregulation of immune checkpoint molecules, such as PD-L1, facilitates immune evasion, further promoting tumor persistence [71].

BRD4 has also been implicated in the DNA damage response (DDR), a multifaceted cellular mechanism that ensures genome stability by detecting DNA lesions, initiating signaling cascades and promoting either repair or apoptosis when damage is irreparable [86]. While this system protects normal cells, in cancer it paradoxically contributes to therapeutic resistance, as efficient repair allows tumor cells to survive genotoxic stress induced by radiation or chemotherapy [87,88]. It has been shown that BRD4 is an important factor in the DDR, acting not only by modulating the transcription of DNA repair genes but also through transcription-independent mechanisms [89,90]. BRD4 facilitates the activation of the non-homologous end joining (NHEJ) pathway and interacts with repair proteins, such as 53BP1, serving as a scaffold that bridges acetylated histones with the DNA repair machinery [89,90,91]. Inhibition or silencing of BRD4 through BET inhibitors (BETis) enhances yH2AX accumulation, leading to persistent DNA damage, genomic instability and cell death. The increased formation of yH2AX foci indicates the presence of unrepaired DNA double-strand breaks, underscoring the impairment of efficient DNA damage resolution upon BRD4 inhibition [89].

Additionally, BRD4 is implicated in replication stress response and DNA damage checkpoint activation via its interaction with the pre-replication complex component the Cell Division Cycle 6 (CDC6). A loss of BRD4 function impairs ATR/CHK1 signaling, sensitizing cancer cells to DNA stress-inducing agents [92]. Interestingly, BRD4 has been reported to act as a negative regulator of the DDR by insulating chromatin from damage singling through recruitment of condensin II components (SMC2, SMC4), thereby promoting chromatin compaction [93].

## 4. Pharmacological Targeting of BRD4

### 4.1. BET Inhibitors

BET inhibitors can be grouped into several pharmacological classes. Pan-BET inhibitors, such as JQ1, bind to both BD1 and BD2 bromodomains, blocking BRD4 and other BET family proteins [58]. Domain-selective inhibitors engage either BD1 or BD2, which allows for the modulation of specific transcriptional programs while reducing systemic toxicity [61,94]. Bivalent inhibitors, like AZD5153, bridge two bromodomains simultaneously, enhancing binding potency and chromatin residence time [95]. Finally, dual/multi-target agents combine BET inhibition with other therapeutic mechanisms, such as HDAC, PARP1, or CBP/p300 inhibition, to increase efficacy and overcome adaptive resistance [96,97]. This section concludes with a summary that integrates all of the inhibitors described (Table 1).

BET inhibitors exert their antitumor effects in GBM primarily by disrupting BRD4-mediated transcriptional regulation, which controls genes associated with proliferation, stemness, and survival. Each pharmacological class of BET inhibitors exhibits distinct mechanistic features and cellular outcomes in a GBM model. JQ1 and other pan-BET inhibitors displace BRD4 from chromatin and suppressive oncogenic transcriptional programs, including *MYC*-driven and super enhancer-associated genes. In GBM cells, this results in cell cycle arrest, inhibition of stem-like features, and induction of apoptosis or senescence depending on the cellular context. JQ1 also suppresses key signaling pathways such as VEGF/PI3K/AKT, limiting the proliferation and invasiveness of glioma stem-like cells [58]. BD1 and BD2 selective inhibitors target individual bromodomains to modulate transcription more selectively. BD1-selective inhibitors often replicate many of the antiproliferative effects of pan-BET inhibitors in GBM models, whereas BD2-selective inhibitors predominantly affect inducible transcriptional programs, such as stress or inflammation-responsive genes, which can influence tumor microenvironment (TME) interactions. These domain-selective inhibitors can reduce systemic toxicity while maintaining efficacy in vitro and in orthotopic GBM models [61,98]. Bivalent inhibitors, exemplified by AZD5153, simultaneously engage two bromodomains within BRD4, resulting in increased chromatin residence and stronger transcriptional suppression. In GBM models, bivalent inhibitors more effectively downregulate oncogenic gene expression and inhibit tumor growth compared with monovalent inhibitors [95]. Dual/multi-target inhibitors, including NEO2734, combine BET inhibition with additional targets such as CBP/p300, HDAQCs, or PARP1. In GBM models, these compounds simultaneously suppress BRD4-dependent transcription and complementary pathways, producing antiproliferative effects, stronger induction of apoptosis, and enhanced tumor growth inhibition. By concurrently targeting multiple epigenetic regulators, these dual inhibitors can also delay or prevent the development of adaptive resistance mechanisms, including enhancer reprogramming and compensatory kinase activation [59,96].

BET inhibitors vary markedly in potency, bromodomain selectivity, bioavailability and toxicity profiles, as well as in their pharmacokinetic (PK) and pharmacodynamic (PD) properties. These differences critically influence their performance in GBM models and their translational potential.

Pan-BET inhibitors exhibit high biochemical potency, but lack domain selectivity, resulting in broad transcriptional suppression [59,61]. In contrast, BD1-selective inhibitors tend to reproduce the antiproliferative effects of pan-BETi by primarily targeting constitutive BRD4 transcriptional output, whereas BD2-selective inhibitors modulate inducible, signal-responsive transcriptional programs, which may decrease systemic toxicity [61,73]. Bivalent inhibitors engage two bromodomains simultaneously, leading to prolonged target residence time and more profound repression of SE-driven genes compared with monovalent inhibitors [95]. Dual-target molecules achieve broader transcriptional repression and enhanced cytotoxicity relative to single-target inhibitors [96].

Limited brain penetration remains the major barrier for BET-targeting strategies in GBM. Most benchmark BET inhibitors are substrates of P-glycoprotein (P-gp), resulting in low intracranial concentrations despite robust in vitro activity [99]. Newer analogues such as UM-002, were optimized for enhanced CNS exposure, exhibiting improved brain-to-plasma ratios, and clear effects in glioma organoids [25]. Bivalent inhibitors often display blood–brain barrier (BBB) permeability due to their increased molecular size and polarity [95].

Pan-BET inhibitors exhibit relatively short target residence times, and transcription can occur rapidly after drug washout, complicating sustained target suppression in vivo [59]. Bivalent inhibitors produce stronger and more durable PD effects, yet their PK properties remain limiting factors [95]. Dual-target inhibitors may present complex PK interactions due to the presence of two pharmacophores, which can affect metabolic stability or cause drug–drug interactions at the level of hepatic enzymes [96].

Hematologic toxicity is the most common adverse effect of pan-BET inhibitors and results from on-target suppression of BRD4 in megakaryocyte progenitors [28]. BD-selective inhibitors may reduce this toxicity by sparing one bromodomain and narrowing the transcriptional repression effect [61,73]. Bivalent inhibitors show a similar but somewhat attenuated hematologic toxicity profile and reduced gastrointestinal toxicity compared to JQ1 [95]. Dual inhibitors frequently exhibit synergistic toxicities, reflecting inhibition of multiple essential epigenetic pathways [100].

Research on BET family proteins, particularly BRD4, relies on both in vitro and in vivo experimental systems, which provide complementary insights but differ substantially in biological complexity and translational relevance. In vitro models have been instrumental in identifying the molecular effects of BET inhibition, such as suppression of *MYC* expression, attenuation of SE activity, and inhibition of oncogenic transcriptional elongation. These systems enable mechanistic precision and high-throughput drug screening but lack key features of tumor biology, including three-dimensional tissue architecture, stromal interactions, immune components, and PK constraints [50,58]. In contrast, in vivo models provide a more physiologically relevant framework to evaluate BET inhibitor efficacy, drug bioavailability, systemic toxicity, and TME interactions. While these models offer improved translational relevance, species-specific differences in epigenetic regulation, metabolism, and immune response limit their ability to fully predict clinical outcomes [59,101].

Despite encouraging in vitro and in vivo efficacy, clinical translation of BET inhibitors has been challenging, with trials reporting modest and often transient responses across multiple cancer types. These limitations reflect adaptive resistance mechanisms, transcriptional rewiring, and dose-limiting toxicities arising from the essential role of BRD4 in normal cellular gene regulation. Moreover, the context-dependent function of BRD4 across tumor subtypes complicates the division of patients into risk groups and reduces the predictive accuracy of preclinical models. Collectively, these findings underscore the need for more predictable translational platforms, including patient-derived organoids, integrative multi-omics profiling and next-generation BET-targeting strategies such as BD1/BD2-selective inhibitors and PROTAC-based BRD4 degraders, which may enhance specificity and therapeutic durability [60,102,103].

### 4.2. BRD4 Degraders

Regardless of the potential of BRD4 inhibitors in cancer therapy, their effectiveness in GBM has been hampered by the accumulation of BRD4 proteins and the development of drug resistance in cancer cells [73]. To overcome these limitations, proteolysis targeting chimeras (PROTACs) have been introduced as a novel therapeutic class that utilizes the ubiquitin–proteasome system to catalyze the elimination of BRD4 rather than merely inhibiting its function [104]. These bifunctional molecules function by simultaneously binding to a specific target protein and an E3 ubiquitin ligase, such as cereblon (CRBN) or von Hippel–Linday (VHL), thereby bringing them into close proximity to induce polyubiquitination and subsequent proteasomal degradation of the target [105,106]. Despite PROTACs offering a transformative approach to protein degradation, their clinical translation for GBM faces considerable obstacles, primarily due to issues regarding high molecular weight and low cell permeability. A summary of the discussed degraders is provided in Table 1 at the end of this section.

Among the developed compounds, dBET6 stands out as a CRBN-recruiting degrader derived from dBET1 that demonstrates the ability to degrade BRD2, BRD3, and BRD4 in a short period of time [29]. Research conducted by Yang et al. [28] indicates that dBET6 not only exhibits significantly better antitumor activity than traditional BET bromodomain inhibitors but also possesses the potential to overcome acquired resistance to these inhibitors in GBM. dBET6 disrupts transcriptional regulation controlled by BET and E2F1 and induces cell cycle arrest at the G2/M phase, which is distinct from the G1 phase arrest typically caused by BRD4 inhibitors.

Another potent degrader, ARV-825, which links the inhibitor OTZ015 with a CRBN ligand, exhibits superior antitumor activity at lower doses compared to occupancy-based inhibitors like JQ1. Comparative analysis [107] shows that ARV-825 is more effective in reducing c-MYC protein levels, inhibiting proliferation, and inducing apoptosis than BET inhibitors (e.g., JQ1 or OTX015).

Moreover, Duan et al. [66] reported that the VHL-dependent pan-BET PROTAC GNE987 was found to inhibit GBM cell proliferation in a dose- and time-dependent manner, exhibiting an IC_50_ (half-maximal inhibitory concentration) of 9.89 nM, significantly lower than that of JQ1 or ARV-825.

The novel degrader ZBC260 has also been evaluated for its efficacy against GBM, demonstrating the capacity to inhibit cell proliferation and induce cell cycle arrest in glioma cell lines. ZBC260 treatment has been shown by Tian et al. [79] to reduce tumor growth and inhibit stem cell-like properties in GBM.

Additionally, the VHL-based PROTAC MZ1, known for its rapid and selective degradation of BRD4 over BRD2 and BRD3, is being investigated to evaluate its specific therapeutic potential in GBM models [47].

Despite the promising antitumor effects, the ability of BRD4 degraders to cross the BBB safely and effectively remains to be fully elucidated, necessitating further development of brain-penetrant molecules that can selectively target tumor cells while sparing healthy brain tissue. Furthermore, since the mechanism of PROTACs depends on intrinsic proteasomal activity, identifying biomarkers capable of predicting therapeutic response will be crucial for the clinical implementation of these therapies in GBM [27].

**Table 1:** Summary of BRD4 inhibitors or degraders as monotherapy in GBM.

Class of BET Inhibitors/Degraders	Representative Compounds	Potency and Bromodomain Selectivity	Brain Penetration/Bioavailability	Toxicity Profile	PK/PD Limitation	References
Pan-BET inhibitors	JQ1, I-BET762, OTX-015	high potency; non-selective broad transcriptional repression	generally poor BBB penetration; many are P-gp substrates	dose-limiting thrombocytopenia; GI toxicity	short residence time; transcriptional rebound; limited CNS AUC	[58,59,100]
BD1-selective inhibitors	GSK778, BD1-biased tool compounds	preferential BD1 inhibition; strong effects on constitutive cancer transcription	variable; BBB penetration often limited but better than that of large bivalent or PROTAC molecules	potentially reduced hematologic toxicity vs. pan-BET	selectivity may reduce efficacy in highly inducible tumors	[61,73]
BD2-selective inhibitors	GSK046, ABBV-744	preferential modulation of inducible transcription; anti-inflammatory bias	limited data, typically modest BBB penetration	generally milder hematologic toxicity	may require combination for full antitumor effect	[61,73]
Bivalent BET inhibitors	AZD5153	dual-bromodomain engagement; very strong BRD4 suppression, prolonged target residence	BBB penetration low due to larger molecular weight	moderate hematologic toxicity; often better tolerated than pan-BET	slow absorption; low tissue distribution; more complex PK	[95]
Dual/multi-target inhibitors	NEO2734 (BET + CBP/p300)	broad-spectrum epigenetic suppression; synergistic effects on oncogenic transcription	PK highly variable; large hybrid molecules often show reduced CNS permeability	additive toxicity (hematologic + epigenetic target-relate)	complex metabolism; dual target interactions may affect dosing	[96]
CNS-optimized BET analogues	UM-002	moderate potency; structure; optimized for BBB penetration	high CNS exposure compared with classical BETi	toxicity not fully characterized; improved brain targeting may reduce systemic dose	PK favorable for brain tumors; PD more sustained in CNS	[28]
PROTAC BET degraders	dBET6	highpotency; targeting BRD2, BRD3 and BRD4; more active than BET inhibitors	specific BBB data are limited; poor cell penetration and high molecular weight	induces G2/M phase cell cycle arrest and overcomes acquired resistance	therapeutic efficacy is dependent on the functional integrity of the tumor’s proteasomal machinery	[28,29]
	ARV-825	targeting BRD2, BRD3 and BRD4; degrades the target protein at sub-nanomolar levels (DC50 < 1 nM) by functioning catalytically at very low doses	poor membrane permeability	high potency at reduced dosages minimizes the risk of drug resistance	PK profiles are constrained by the large size and potential instability of the heterobifunctional structure	[29,106,108]
	GNE987	VHL-dependent; targeting BRD2, BRD3 and BRD4; IC50 = 9.89 nM in U87 cells (outperforming JQ1 and ARV-825)	specific BBB data are limited; physicochemical properties hinder effective delivery to intracranial tumors	exerts antiproliferative effects in a dose- and time-dependent manner	requires PK optimization to overcome delivery barriers associated with GBM	[66]
	ZBC260	potent degrader; targeting BRD2, BRD3 and BRD4; kills cancer cells more efficiently than JQ1	systemic bioavailability is suggested by in vivo efficacy; BBB penetration remains a challenge	well-tolerated in murine models; specifically suppresses stem cell-like traits by repressing the Wnt/β-catenin signaling pathway	biological activity relies on specific E3 ligase recruitment and ubiquitin–proteasome system function	[78,109]
	MZ1	displays isoform selectively, preferentially degrading BRD4 over BRD2/3 (dose dependent) via cooperative ternary complex formation	N/A ^1^	selectivity offers a potentially improved safety profile	the degradation effect is reversible, requiring sustained exposure to maintain effective protein depletion	[47,110]

^1^ N/A: Information not avaible.

## 5. Therapeutic Resistance and Limitations of BRD4 Inhibition in GBM

Despite the promising therapeutic potential of BET bromodomain inhibitors targeting BRD4 in GBM, their clinical efficacy remains limited. Growing evidence indicates that resistance mechanisms, together with pharmacological barriers such as insufficient BBB penetration, substantially constrain therapeutic outcomes. Addressing these challenges is essential for optimizing BRD4-targeted strategies and developing more effective combinatorial or next-generation therapies for GBM.

Recent studies indicate that inhibition of BRD4 can induce compensatory overexpression of BRD2 and BRD3, two closely related BET family proteins, which partially restore chromatin occupancy and maintain the transcription of survival-promoting genes [111]. This functional redundancy within the BET family reduces the sustained antitumor effects of BRD4-selective inhibitors and underscores the necessity of approaches that either simultaneously target multiple BET proteins or circumvent the compensatory mechanisms mediated by BRD2 [29,111]. Notably, despite its compensatory upregulation, BRD3 does not appear to play a major role in adaptive resistance in GBM, as its knockdown produces minimal effects on GBM cell proliferation [98,112].

Evidence demonstrates that BRD2 upregulation is a conserved, frequently observed adaptive response to BET inhibition across multiple cancer types, in which increased levels function to sustain essential transcriptional programs despite the displacement of BRD4 [111]. Mechanistically, this feedback loop can be driven by the transcription factor NEYA, which directly occupies the BRD2 promoter and mediates its transcriptional induction following treatment with inhibitors such as JQ1 [111]. Consistent with paralog compensation, BRD2 and BRD4 exhibit largely overlapping genomic occupancy, allowing BRD2 to partially maintain transcription of select target genes after BRD4 eviction [113].

In GBM, BRD2 specifically facilitates cell state plasticity by regulating mesenchymal gene expression through NF-κB signaling in mesenchymal-like cells, thereby modulating therapeutic resistance [113]. Although the initial response to BET inhibition often involves the suppression of key oncogenes such as *MYC*, resistant cells frequently exhibit a rapid rebound of these transcripts and other survival programs [114,115]. Importantly, this transcriptional reprogramming is not merely a passive consequence of BRD4 loss but involves active remodeling of the regulatory landscape, including the redistribution of transcriptional machinery and enhancement in context-dependent alternative regulatory circuits at genomic regions previously bound by BRD4 [114,115].

The study by Han et al. [116] identifies several mechanisms underlying GBM resistance to BRD4 inhibition. Although JQ1 initially suppresses tumor growth, GBM cell lines display variable sensitivity, and some tumors exhibit rapid regrowth following transient exposure. A central finding is that autophagic degradation of HEXIM1, a negative regulator of the P-TEFb complex, diminishes the effectiveness of BRD4 inhibition. Loss of HEXIM1 enhances BRD4-driven transcriptional activity and reduces responsiveness to JQ1, a process further modulated by PI3K/AKT signaling. In particular, pharmacological inhibition of autophagy and the AKT pathway using ubenimex restores HEXIM1 levels, resensitizes GBM cells to JQ1 and suppresses tumor proliferation and migration. These observations indicate that autophagy-mediated HEXIM1 degradation and AKT activation contribute to BET inhibitor resistance and represent viable targets for combination therapy.

Based on the findings of Čančer et al. [117], GBM exhibits pronounced heterogeneity that drives variable sensitivity to BRD4 inhibition. While JQ1 induces apoptosis and suppresses proliferation in cells dependent on *MYCN* and Aurora kinase A (AURKA), a substantial subset of tumors remains resistant. These resistant cells rely on *MYCN*-independent survival programs, activate DNA repair pathways, and maintain cell cycle progression, thereby limiting the cytotoxic impact of BRD4 inhibition. Moreover, resistant GBM cultures often display mesenchymal transcriptional signatures and pro-survival signaling linked to epithelial–mesenchymal transition and hypoxia. Importantly, co-targeting AURKA restores therapeutic sensitivity even in resistant GBM models, underscoring that BRD4 inhibitor resistance is multifactorial and driven by adaptive survival mechanisms requiring rational combination strategies.

Jermakowicz et al. [118] demonstrated that GBM rapidly develops resistance to BRD4 inhibition, thereby limiting the therapeutic efficacy of BET inhibitors. In preclinical GBM models, short-term BETi treatment induced rapid kinome reprogramming within hours, characterized by increased activation of multiple receptor tyrosine kinases, including Ephrin receptors, IGFR1, EGFR, and notably FGFR1. Phosphoproteomic analyses identified FGFR1 among the earliest and most prominently upregulated signaling nodes following BETi treatment. Notably, FGFR1 protein levels increased without corresponding changes in mRNA expression, suggesting a post-transcriptional regulatory mechanism and leading to enhanced MAPK and JNK signaling associated with pro-survival pathways. Importantly, both pharmacological inhibition and siRNA-mediated depletion of FGFR1, when combined with BETis, resulted in greater reduction in GBM cell proliferation in vitro and tumor growth in vivo compared to BETis alone. These findings indicate that FGFR1 contributes to the early adaptive signaling response to BETis in GBM and supports the potential benefit of combinatorial therapeutic strategies targeting both BET proteins and FGFR1.

Similar adaptive kinome reprogramming contributing to BETi resistance has been observed in other cancer types, where it involves coordinated activation of multiple receptor tyrosine kinases, rather than being limited to FGFR1, suggesting that this mechanism is broadly conserved across tumors [119,120,121].

Non-coding RNAs (ncRNAs), including miRNAs, lncRNAs, snoRNAs, piRNAs and cricRNAs, are critical regulators of GBM biology, modulating gene expression at transcriptional, post-transcriptional and epigenetic levels [122]. Among these, long non-coding RNAs (lncRNAs) are frequently dysregulated in GBM and drive tumor proliferation, survival and therapy resistance, with HOTAIR, H19 and HOTAIRM1 notably overexpressed in tumor tissue [123,124,125,126,127]. LncRNAs are transcripts longer than 200 nucleotides that regulate gene expression by guiding chromatin-modifying complexes, scaffolding molecular interactions, or modulating other RNAs. When dysregulated, they enhance GBM progression and treatment resistance through activation of oncogenic pathways such as β-catenin and Notch signaling [128].

BET proteins, particularly BRD4, regulate that expression of oncogenic lncRNAs by binding to their regulatory regions, as demonstrated for HOTAIR, linking BRD4 activity to lncRNA-mediated tumorigenic programs [129]. Pharmacological inhibition of BRD4 with inhibitors such as I-BET151 suppresses HOTAIR expression and restores the levels of several lncRNAs downregulated in GBM, including MEG3, NEAT1 and DGCR5, leading to reduced cell proliferation and enhanced apoptosis in preclinical models [129].

Other ncRNAs, such as cricRNAs, also interact with BRD4-regulated pathways, acting as miRNA “sponges” and modulating oncogenic signaling [122]. These findings underscore that BRD4 exerts its pro-tumorigenic effects in part through ncRNA networks, highlighting lncRNA modulation as a key mechanism of BET inhibitors’ efficacy in GBM.

The BBB tightly regulates the exchanges of oxygen and metabolites between the blood and the brain, creating an immune-privileged environment by blocking neurotoxic agents and pathogens while also limiting the delivery of many therapeutic agents, which represents a major obstacle in GBM therapy [130,131]. Effective CNS distribution of small-molecule inhibitors is particularly critical for GBM-directed therapeutics, as all GBMs contain regions with a relatively intact BBB [132]. While some BRD4 inhibitors exhibit sufficient brain penetration, such as the clinically promising BET inhibitor trotabresib, others, including I-BET151, show limited BBB permeability, partly due to their high polar surface, which can reduce their in vivo efficacy [98,133]. Optimizing chemical properties is therefore essential, particularly for BRD4 degraders, which are prone to exclusion from the brain because of their large size. Future efforts should focus on developing smaller, brain-penetrant degraders of BET proteins [27].

Sang et al. [134] showed that the BBB significantly restricts CNS delivery of therapeutic agents, including BRD4 inhibitors. To address this, a specialized nanoplatform functionalized with an Apolipoprotein E (ApoE) peptide was designed to deliver BRD4 siRNA across the BBB via receptor-mediated transcytosis through low-density lipoprotein receptors (LDLRs) on brain endothelial cells. This strategy enables efficient BRD4 silencing within the TME, inducing tumor cell apoptosis, and downregulating the immunosuppressive checkpoint molecule PD-L1. Thus, targeted nanodelivery represents a promising approach to enhance the efficacy of BRD4-targeting therapies by overcoming the BBB barrier.

Sex-related differences also influence both the incidence and prognosis of GBM [135,136]. According to Kfoury et al. [137], GBM cells exhibit a pronounced sex-specific response to BET inhibitors, suggesting that biological sex differences in the disease directly impact therapeutic outcomes. Male and female GBM cells show differential sensitivity to both pharmacological and genetic inhibition of BRD4. Specifically, male GBM cells are markedly more responsive to BET inhibitors, such as JQ1 or CPI0610, exhibiting reduced proliferation and increased cell death. In these cells, BRD4 inhibition or knockdown consistently decreased clonogenic potential and in vivo tumorigenicity. In contrast, female GBM cells responded differently, with BRD4 inhibition leading to increased clonogenic frequency in vitro and a higher likelihood of tumor formation in vivo. Consequently, sex should be taken into account when applying BRD4-targeted therapies, including inhibitors and degrades, in GBM patients.

## 6. Clinical Applications of BRD4 Targeting in GBM

Despite the strong anti-GBM effects of BRD4 inhibition observed in preclinical studies, BRD4 inhibitors have yet to be successfully translated into clinical practice. Nevertheless, BET inhibitors have recently emerged as promising epigenetic therapeutics for GBM [27]. Among these, trotabresib (CC-90010) has been investigated in early-phase clinical trials involving patients with high-grade gliomas (NCT04047303) [133]. The primary objectives of these studies were to evaluate the compound’s safety, tolerability, PK, PD, and ability to penetrate tumor tissue. Initial findings suggest that trotabresib is generally well tolerated, although further refinement of dosing regimens is needed to enhance tumor exposure and therapeutic efficacy [133]. Moreover, trotabresib administered in combination with TMZ, or concurrently with TMZ and RT, has also shown tolerability in newly diagnosed and recurrent GBM patients (NCT04324840).

While these results highlight the promise of trotabresib as a clinically viable BET inhibitor, translating other BRD4-targeting agents into effective therapies for GBM remains challenging. For instance, JQ1’s clinical utility is limited by its very short half-life—approximately one hour after administration [58,138]. Similarly, the development of BRD4 degraders for clinical use is further complicated by suboptimal physicochemical properties and low bioavailability, largely attributable to their sizable molecular weight and small E3 ligands [73].

Given the multifactorial nature of GBM and the limited efficacy of BRD4 inhibitor monotherapy, combination strategies have become a central focus in developing BRD4-targeted treatments [139]. Although compounds such as (+)− JQ1 demonstrate promising antitumor activity in preclinical models, their clinical utility as single agents is hindered by adaptive tumor mechanisms, including altered BRD4 stability, post-translational modifications and activation of compensatory pathways. Dose-limiting toxicities further restrict the therapeutic window, underscoring the challenges of monotherapy [139].

Consequently, ongoing and planned studies are evaluating approaches that integrate BRD4 inhibitors with both established and emerging therapeutic modalities, aiming to enhance efficacy and achieve more durable anticancer responses. These investigations will be instrumental in defining the clinical relevance of targeting BRD4 in GBM. An overall summary of all the combined approaches described in this section is provided in Table 2.

### 6.1. BRD4 Inhibition + TMZ

The current standard of care for patients with GBM includes chemotherapy with TMZ, an alkylating agent that includes DNA damage. Inhibition of BET proteins with the small-molecule compound JQ1 has been shown to influence the cellular response to DNA damage in several cancer cell lines in vitro and to promote arrest in the G1 phase of the cell cycle as well as a reduction in mitochondrial membrane potential. Importantly, suppression of BET bromodomain proteins has also been reported to exert antitumor effects in mouse models of glioma [140].

Colorado et al. [25] demonstrates that pharmacological inhibition of BET proteins with JQ1 markedly enhances the therapeutic efficacy of TMZ in GBM models. While JQ1 alone reduces cell proliferation by inducing cell cycle arrest and promoting a shift toward a differentiated, neuronal-like phenotype, its co-administration with TMZ leads to a substantially stronger induction of apoptosis in GBM cells. This synergistic effect arises because JQ1 not only sensitizes tumor cells to TMZ-mediated cytotoxicity but also diminishes stem-like properties that typically contribute to chemoresistance. The data therefore suggest that combining BET inhibition with standard TMZ treatment may represent a promising strategy to overcome intrinsic resistance mechanisms and improve therapeutic responses in GBM.

The combination of OTX015 and TMZ represents a potent synergistic strategy to overcome drug resistance and improve therapeutics outcomes in GBM [25,140]. OTX015 enhances TMZ sensitivity by inhibiting BRD4, thereby impairing DNA damage repair—one of the main mechanisms underlying TMZ resistance [140]. In addition, OTX015 exerts independent antitumor effects by inducing cell cycle arrest [25,140].

The study by Berenguer-Daizé [141] showed that in vitro experiments in U87MG cells revealed synergistic to additive effects, with the strongest synergy occurring when OTX015 was administered prior to TMZ. In vivo, simultaneous oral administration of both agents in an orthoptic U87MG model significantly prolonged median survival (49 vs. 16.5 days) without inducing the bodyweight loss typically associated with TMZ monotherapy. Moreover, OTX015 was shown to cross the BBB efficiently and accumulate in tumor tissue at concentrations 7–15 times higher than in normal brain tissue.

According to Guo et al. [142], combining BRD4-targeting PROTACs, such as ARV-825, with TMZ produces synergistic antitumor effects in GBM cells. The study demonstrates that nanoparticle-mediated delivery overcomes limitations of PROTACs, including poor solubility and bioavailability, enhancing their therapeutic potential. These findings indicate that co-administration of PROTACs with TMZ, particularly via optimized nanovehicles, represents a promising strategy to translate catalytic protein degradation into clinically viable GBM treatments.

### 6.2. BRD4 Inhibition + Radiotherapy 

Concomitant RT with TMZ followed by adjuvant TMZ, together with surgical intervention, represents the standard therapeutic approach for patients with newly diagnosed GBM. Nonetheless, the overall effectiveness of radiation remains constrained by the intrinsic resistance of these tumors [27].

Fan et al. [69] showed that combining RT with the BRD4 inhibitor I-BET151 produces strong synergistic effects, exceeding the impact of either treatment alone and providing a rationale for overcoming acquired radioresistance. Mechanistically, this radiosensitization is driven by the post-radiation upregulation of BRD4, a phenomenon observed in recurrent GBM tumors and correlated with poor prognosis in patients receiving RT-based therapy. A key component of this process is the inhibition of SE-driven transcription of the *COL1A1* gene, a major constituent of the extracellular matrix (ECM). By suppressing this BRD4/*COL1A1* axis, the combined treatment not only reduces cellular proliferation, promotes apoptosis and increases persistent DNA damage in vitro, but also beneficially reprograms the TME. This TME remodeling includes decreased inflation of immunosuppressive MS macrophages and neutrophils, coupled with increased accumulation of antitumor CD8+ T cells in GBM tissue following irradiation.

### 6.3. BRD4 Inhibition + PDT

The synergistic strategy of BRD4 inhibition combined with nonionizing photodynamic therapy (PDT) offers a potent mechanism to enhance the cytotoxic effects against GBM [143,144]. PDT’s efficacy in GBM is often counteracted by an innate resistance mechanism where therapy-induced stress promotes an aggressive, proliferative, and invasive phenotype in surviving cells. This is mediated by the upregulation of inducible nitric oxide synthase (iNOS) and the resulting generation of nitric oxide (NO), which facilitates resistance to apoptosis [143,145]. At the molecular level, iNOS transcription is critically regulated by the cooperative action of the transcription factor NF-κB and the BET protein BRD4. Specifically, BRD4 serves as a key co-activator by binding to acetylated lysine residues on NF-κB p65 [143].

In a study by Fahey et al. [143], pharmacological intervention with JQ1 effectively disrupted this pro-survival signaling cascade. JQ1 strengthened PDT responses by directly interfering with the BRD4–NF-κB interaction, thereby suppressing the NF-κB-dependent expression of resistance genes, including iNOS, survivin and Bcl-xL. This suppression mitigated NO generation and curtailed the heightened aggressiveness and proliferation of GBM cells following PDT-induced stress, as demonstrated in U87 cell cultures [143,144]. Preclinical evidence supports this combined approach, showing that the addition of JQ1 resulted in a superior cytotoxic outcome, reducing U87 cell viability to approximately 45%, a level significantly lower than that achieved by either agent alone. Consequently, targeting iNOS expression through BRD4 inhibition represents a rational molecular strategy to overcome inherent treatment antagonism and substantially improve the therapeutic efficacy of PDT in GBM.

### 6.4. BRD4 Inhibition + Immunotherapy

The therapeutic effectiveness of combining BRD4 inhibitors with immunotherapy in GBM is centrally underpinned by the suppression of the PD-L1 axis, a critical mediator of tumor immune escape [71,146]. BRD4 acts as a key transcriptional regulator of PD-L1, directly binding to its promoter, as shown by ChIP assays, and BET inhibition with JQ1 reduces CD274/PD-L1 expression at both mRNA and protein levels in GBM [16]. Beyond PD-L1 regulation, BRD4 shapes the immune microenvironment by promoting macrophage infiltration and M2 polarization, partly via IRF4. Targeted degradation of BRD4 with ARV-825 in GL261 and U87 models decreased M2 polarization and inhibited STAT3, STAT6 and AKT signaling, highlighting its potential to enhance immune checkpoint blockade [147,148].

**Table 2:** Summary of therapeutic combinations with BRD4 inhibition in GBM.

Combination	Animal, Cell/CI	Therapeutic Regimen	Mechanism	Effects	References
BRD4 inhibition + TMZ					
TMZ + JQ1	U87 and GH2 GBM cells	0.5 µM JQ1 + 500 µM TMZ co-administration	JQ1 inhibits BET proteins, induces differentiation & autophagy, sensitizing cells to TMZ	↑ apoptosis; ↑ anti-proliferative effect vs. TMZ alone	[25]
TMZ + OTX015	U87MG cells; CI ≈ 0.7 (synergy)	OTX015 given prior to or concomitantly with TMZ; also tested simultaneously in mice	BET inhibition, cell cycle arrest, transcriptional modulation sensitizing cells to TMZ	↑ survival in mice; synergistic or additive inhibition of proliferation; no added toxicity	[141]
TMZ + ARV-825 (PROTACs)	G422 glioma cells; CI < 0.75 (synergy across most dose ratios)	ARV-825 in polymer nanovehicles + TMZ	BRD4 degradation increases sensitivity to TMZ-induced DNA damage	↑ γ-H2AX; ↑ apoptosis and cytotoxicity; strong synergistic viability inhibition	[142]
BRD4 inhibition + RT					
RT + I-BET 151	GBM cells (T98G, U87) and GL261 mouse models (in vitro/in vivo)	In vivo: I-BET 151 (10 mg/kg, daily combined with fractionated RT (2 Gy\times 5 days) In vitro: I-BET 151 (µM range) + RT (2.5–7.5 Gy)	Upregulated BRD4 promotes resistance; I-BET 151 suppresses SE-driven *COL1A1* (ECM component); remodeling of TME	↑ prolonged survival; ↑ tumor reduction; ↑ apoptosis; ↑ favorable immunomodulation	[69]
BRD4 inhibition + PDT					
PDT + JQ1	U87 (human cell line)	Cells were treated with increasing concentrations of JQ1, up to 0.5 µM, and then exposed to light irradiation with fluence of approximately 1 Jcm^2^	PDT-induced stress upregulates iNOS/NO in U87 cells, promoting resistance; this is mediated by BRD4 acting as an NF-κB co-activator; JQ1 suppresses this BRD4 function	↓ NO antagonism; ↑ antitumor effects; ↓ resistance to apoptosis; ↓ proliferation and invasiveness	[143]
BRD4 inhibition + immunotherapy					
EGFR CAR-T + JQ1	U87MG, LN229 cells; PDX mouse models	EGFR CAR-T with 150 µM JQ1 for 48 h or 72 h	CAR-T resistance in GBM is driven by the activation of immunosuppressive genes (PD-L1, IDO1) in the TME; BRD4 is required for their transcription, which is blocked by JQ1	↑ extender survival; ↑ significant tumor reduction; ↓ overcomes CAR-T resistance; ↓ mitigates TME immunosuppression	[146]

*EGFR* amplification and/or mutation occurs in nearly 40% of GBM cases, underscoring its relevance as a therapeutic target [27]. Research by Xia et al. [146] into cell-based therapies, specifically *EGFR*-targeted Chimeric Antigen Receptor T cells (CAR-T), revealed that GBM tumors rapidly develop resistance, which is linked to a compensatory immunosuppressive response within the TME involving in situ activation of a comprehensive panel of immunosuppressive genes. Critically, the transcription of this gene cohort has been determined to be dependent on BRD4 activity.

Pharmacological targeting of this dependency using the BRD4 inhibitor JQ1 was shown to block the activation of these key immunosuppressive genes. This epigenetic intervention, when combined with EGFR CAR-T cells, demonstrated significant therapeutic synergy, resulting in suppressed tumor growth and extended overall survival in murine GBM models. These findings collectively position BRD4 inhibition as an effective strategy to counteract the immune-evasive phenotype and enhance the efficacy of T cell-based immunotherapies against GBM [146].

### 6.5. Future Perspectives for Multimodal Therapies

The future of multimodal BRD4 inhibitor applications in GBM is expected to involve more sophisticated integration with established standard-of-care therapies, specifically RT and TMZ. Emerging preclinical evidence suggests that BRD4 inhibition fundamentally alters transcriptional responses to cytotoxic stress, creating temporal windows of enhanced vulnerability that may be missed by simultaneous drug administration [69,70]. Therefore, the research focus has shifted toward strategic sequencing, utilizing BRD4 inhibitors either to prime tumor cells before DNA-damaging treatment or to suppress DNA repair mechanisms during the post-treatment recovery phase.

A recent study by Peng et al. [149] demonstrated that a sequential combination of PARP inhibitors (PARPis) followed by BRD4 inhibitors significantly enhances antitumor efficacy in GBM models while concurrently reducing systemic toxicity. In this preclinical paradigm, the DNA damage initially induced by PARPis persists after the treatment course, which subsequently allows BET inhibition to effectively impair homologous recombination (HR) and selectively target malignant cells. This strategic, sequential combination treatment achieved comparable efficacy to concurrent administration but with improved tolerability. Such findings support the development of a mechanistically guided framework for stratification based on biomarkers associated with DNA repair or replication stress.

Future clinical development will likely employ adaptive trial designs to simultaneously evaluate multiple sequencing strategies. This approach is critical for rapid identification of combinations that maximize therapeutic synergy while minimizing overlapping toxicities. Concurrently, a major priority remains in the development of precision-driven enrollment strategies. The identification of predictive biomarkers, such as those related to transcriptional dependencies, *MGMT* regulation, immune signaling and DNA repair capacity, will be essential for selecting patients most likely to benefit from BRD4-based combination therapies [70,71].

## 7. Future Perspectives and Challenges

Targeting BRD4 in GBM stands at the crossroads of chemical innovation and biological complexity. Although extensive evidence supports the notion that BET inhibition can dismantle key oncogenic transcriptional programs, translating these insights into effective therapies has proven challenging [30,45,59,61]. Limitations related to selectivity, bioavailability, drug resistance, and systemic toxicity continue to restrict clinical success [29,45]. Future progress will require a deeper understanding of BRD4’s cell-specific functions and the development of molecular tools capable of engaging them with surgical precision rather than global blockade [150].

Enhancing selectivity and optimizing pharmacological behavior remain at the core of ongoing BET-targeting drug development. Early inhibitors, such as JQ1 and OTX015, were instrumental in validating BRD4 as a therapeutic target; however, their pan-BET activity, encompassing both BD1 and BD2 domains, was associated with adverse hematologic effects. Therefore, newer generations of agents have been designed with refined selectivity, targeting either specific bromodomains or particular BET family members that drive tumor growth [151,152,153,154]. Such domain-focused molecules, particularly those with BD1 or BD2-bias, attempt to dissociate malignant transcriptional signaling from essential physiological functions, potentially improving metabolic stability and brain penetration-essential attributes for GBM therapy [150,155]. Adjusting lipophilicity, mitigating P-glycoprotein-mediated efflux and balancing polarity for efficient BBB transport are central strategies to secure effective drug concentrations in the tumor microenvironment [156,157]. Moreover, nanotechnology-based delivery systems are being explored to facilitate the CNS delivery of BET inhibitors or BRD4 degraders, thereby overcoming BBB limitations of many epigenetic agents [158].

Nevertheless, increased selectivity alone rarely ensures sustained therapeutic benefit. GBM’s extraordinary plasticity enables rapid adaptation through activation of parallel signaling or epigenetic pathways. Inhibition of BRD4 can provoke regulation of receptor tyrosine kinases, chromatin remodelers, or transcriptional co-activators such as CBP/p300, effectively reinstating oncogenic transcription [118,150]. Addressing this problem will likely demand rationally designed combination or multi-target approaches, coupling BET inhibitors with agents that block adaptive circuits such as FGFR, PI3K/AKT or DNA repair pathways [118,159].

Another challenge to resolve is the potential compensatory upregulation of other BET family members, such as BRD2 and BRD3, following BRD4 downregulation, which may limit therapeutic efficacy and promote the development of treatment resistance. Future studies should aim to better understand the mechanisms regulating this adaptive response and to develop therapeutic strategies that simultaneously modulate the activity of multiple BET family members or combine BET inhibition with other interventions. Additionally, the identification of biomarkers enabling early detection of compensatory expression could facilitate a more personalized approach to therapy, while the development of next-generation inhibitors with broader specificity or selective modulation of downstream effectors may improve the durability and effectiveness of treatment [43,111,121,160,161,162].

Meanwhile, mitigating systemic toxicity remains a pressing objective. Approaches including pulsatile dosing, localized delivery systems, or domain-restricted inhibitors could help sustain efficacy while minimizing adverse effects [28].

Another essential direction involves identifying biomarkers that predict which patients will benefit most from BRD4-directed therapies. The pattern of enhancer activity in GBM varies substantially between tumors, and only a proportion of cases rely on BRD4-dependent SEs that sustain transcription factors such as MYC, SOX2 or OLIG2 [67,129,137]. In addition, circulating tumor DNA and exosomal RNA represent promising non-invasive biomarkers for real-time monitoring of drug activity and emerging resistance [151,152,153,154].

Rapid advances in next-generation technologies are also emerging. PROTAC-based degraders exemplify a model shift: rather than merely inhibiting BRD4, these bifunctional molecules engage E3 ubiquitin ligases to eliminate it altogether [150]. Domain-selective PROTACs now allow for selective degradation of individual bromodomains or BET members, offering a route to minimize off-target toxicity [67,150,163]. In parallel, hybrid or dual-function molecules that couple BRD4 targeting with inhibition of synergistic epigenetic regulators—such as PARP1, HDAC or CBP/p300—represent an emerging class of multi-target therapeutics [118,159].

Concurrently, artificial intelligence (AI) and computational modeling are transforming BRD4 drug discovery. Machine learning frameworks and generative algorithms are now capable of predicting ligand–target interactions, domain selectivity, and BBB permeability with unprecedented accuracy. Virtual screening and molecular dynamics simulations help prioritize compounds before synthesis, while network-based modeling of BRD4-regulated transcription can forecast tumor adaptation and resistance mechanisms. Integrating these computational tools with experimental validation promises to shorten development timelines and align chemical innovation with the biological reality of GBM [69,144,150,151].

## 8. Conclusions

Focusing on BET proteins, BRD4 has emerged as a promising epigenetic strategy for GBM due to its role in regulating oncogenic transcription, therapy resistance, and the stem-like characteristics of tumor cells. Preclinical studies have demonstrated that BRD4 inhibitors, including JQ1, OTX015 and CPI-0610, can attenuate tumor growth, modulate the TME, and enhance the sensitivity of tumor cells to RT, TMZ chemotherapy, and targeted therapies. Nevertheless, their clinical efficacy has been limited by reversible binding, partial target inhibition, and the rapid onset of resistance. The development of BET degraders, notably PROTAC-based molecules such as dBET6, ARV-825, GNE987 and ZBC260, provides a more durable therapeutic approach by promoting the catalytic elimination of BET proteins rather than merely inhibiting their activity. These degraders enable profound suppression of oncogenic signaling pathways, prevent compensatory accumulation of BRD4, and exhibit efficacy against GSCs.

Despite these encouraging findings, significant challenges persist, particularly with respect to achieving effective BBB penetration and optimizing PK properties for CNS delivery. Future progress will necessitate the development of BBB-permeable degraders, identification of predictive biomarkers, and rational incorporation of BET-targeted agents into combination regimens. In summary, while BET degradation represents a significant conceptual and therapeutic advancement over classical inhibition, its successful clinical translation in GBM will depend upon overcoming delivery obstacles and integrating molecular insights with personalized treatment strategies.

## Data Availability

No new data was created or analyzed in this study. Data sharing is not applicable to this article.
